# Challenging management of gingival squamous cell carcinoma: a 10 years single-center retrospective study on Northern-Italian patients

**DOI:** 10.4317/medoral.23913

**Published:** 2020-08-27

**Authors:** Paolo G. Arduino, Mario Carbone, Alessio Gambino, Marco Cabras, Filippo Cannarsa, Alessandra Macciotta, Davide Conrotto, Roberto Broccoletti

**Affiliations:** 1 Department of Surgical Sciences, CIR-Dental School, University of Turin, Turin, Italy; 2 Department of Clinical and Biological Sciences, University of Turin, Turin, Italy

## Abstract

**Background:**

Aim of this study was to describe the outcome of patients with gingival squamous cell carcinoma (GSCC), and to recognize aspects affecting clinical course and to consider survival rate.

**Material and Methods:**

The case records of patients, over a 10-year period, were retrospectively examined. Differences in distribution of the potential risk factors by prognosis were investigated through non-parametrical tests (Wilcoxon Rank-Sum and Fisher’s Exact). Survival curves for age, therapy and stage were built by the Kaplan-Meier method and compared with Log-Rank test.

**Results:**

79 patients were analysed. Significant increase in mortality for patients older than 77 and for those with advanced stages was found. Cumulative survival rate 5 years after the diagnosis was 43%, while at 10 years was of 11%.

**Conclusions:**

With a statistical relationship between age and tumour stage with survival rates, and 70% of GSCC cases identified as stage IV, early GSCC diagnosis remains challenging.

** Key words:**Gingival cancer, clinical appearance, treatment, retrospective study.

## Introduction

Oral squamous cell carcinoma (OSCC) is the most common head and neck cancer, accounting for more than 90% of total cases; between them, gingival squamous cell carcinoma (GSCC) is a rare disease, most commonly affecting elderly population (1,2). It represents usually 10% of all oral cancers in Europe and the United States, but in Japan, it is only second to tongue cancer, which remains the most common subset (3). Data about GSCC from Italy are lacking; last decade we reported one of the biggest case series of oral cancer, describing a prevalence of gingival involvement in about less than 15% of total cases (4).

When separating the oral cavity into subgroups, statistical power could be lost in the final evaluation, justifying why authors often group the subsites together into ‘‘oral cancer’’; this pooling usually gives fewer data about the unrelated trends and possible prognosis on the subsite levels (5).

Despite the significant advances obtained in the diagnostic and therapeutic opportunities, OSCC continues to portend a modest prognosis with an estimated 5-year overall survival rate of 56% in Western Europe (1). TNM stage, the grade and the depth of invasion of the tumour seem to have still an important effect on the course of the disease; nevertheless, summarizing the survey of the available literature data, it may be stated that the prognostic value of the classical clinicopathologic parameters is ambiguous (6).

Recent data about gingival cancer reported a very bad prognosis (7,8), but Italian data are actually missing. The purpose of this single-center retrospective study was to analyse the outcome of Northern Italian patients with GSCC, treated by distinctive modalities, and to report factors that may have affected survival rate.

## Material and Methods

From a standardised computerised database, medical charts of patients, with histologically confirmed diagnosis of GSCC, at the Oral Medicine Section (CIR-Dental School, Turin), over a 10-year period from June 1, 2008 to June 1, 2018, were retrospectively examined. Subjects were resident in Piedmont region, North-west Italy.

Inclusion criteria were the following: patients with histopathologic diagnosis of GSCC, formulated since June 1st 2008, emerging in gingiva, and biopsied either in gingiva, or in edentulous ridge, or in the retromolar area/tuber maxillae, providing complete information on sex/age of the patient involved, TNM staging, and subsequent follow-up, including exitus, whenever occurred.

Exclusion criteria were the following: incomplete data on age/sex or follow-up of patients with GSCC; lack of histopathologic record of GSCC; lack of TNM staging; histopathologic diagnosis of GSCC before June 1st 2008; diagnosis of malignancies other than GSCC, such as verrucous carcinoma, salivary gland or mesenchymal malignancy.

Demographic information, smoking (current or former smoker vs. non-smoker), alcohol consumption (current of former drinker vs. non-drinker), history of oral potentially malignant disorders (OPMD), tumour site, tumour grade according to the WHO classification (well, moderately or poorly differentiated), T classification and neck nodes involvement at the time of diagnosis (UICC, 1997), staging based on the American Joint Committee on Cancer staging system (7th edition) (9), in situ hybridization (ISH) for HPV detection performed on formalin-fixed, paraffin-embedded tissue using the Bond TM Ready-to-Use ISH HPV Probe (Leica Biosystems, Newcastle, UK) for the following subtypes: 16, 18, 31, 33, 51. ISH was carried out following the manufacturer's instructions on the automated Leica BOND system (BOND-MAX, Leica Biosystems). Furthermore, treatment protocols (surgery, chemotherapy, radiotherapy, either alone or combined) outcome and survival rate were also reviewed. We evaluated patient survival as of August 31st, 2019.

Typical follow up schedule was the same as that already reported (4).

A descriptive analysis was performed. Continuous variables were expressed as median and interquartile range, categorical variables as frequencies and percentages. Non-parametrical tests (Wilcoxon Rank-Sum and Fisher’s Exact tests for continuous and categorical variables, respectively) were used in order to analyse differences in distribution of the variables listed above by the prognosis. Kaplan-Meier method and Log-Rank test were used in order to estimate and compare survivals in different classes of age, therapy and stage, separately. All statistical analyses were performed using R software (version 3.5.3). Statistical significance was defined at *P* value of ≤0.05.

## Results

Finally, 85 cases of GSCC were collected. With 6 clinical charts providing insufficient clinical and/or histopathologic data, 79 cases of GSCC were analysed, in 36 men (mean age: 67.5 ± 10.6) years and 43 women (mean age: 73.49 ± 11.1) (Table 1), with GSCC.

Of these, 21 had previous OPMD, of which 8 (5 F;3 M) Oral Lichen Planus, 7 (7 F) with proliferative verrucous leukoplakia, and 6 (2 F; 4 M) with oral leukoplakia.

Almost 70% of total cases were identified with an advanced stage (54 patients with stage IV). Histologically, 21 cases were identified as well differentiated, 48 as moderately differentiated and 10 as poorly differentiated. The mandible was more commonly affected (69.6%).

Almost 65% of the reported patients did not report the classical risk factors for oral cancer (e.g. tobacco and alcohol) (Table 1). In situ hybridization (ISH) for HPV revealed 5 cases resulted positive to HPV (Table 2).

**Table 1:**
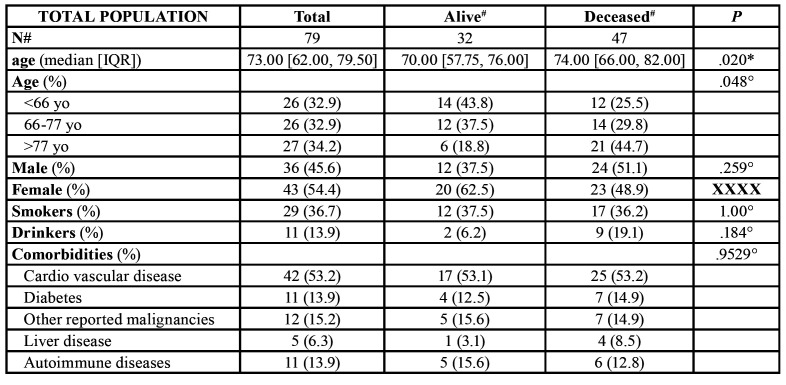
Main characteristics of patients with GSCCs.

**Table 2:**
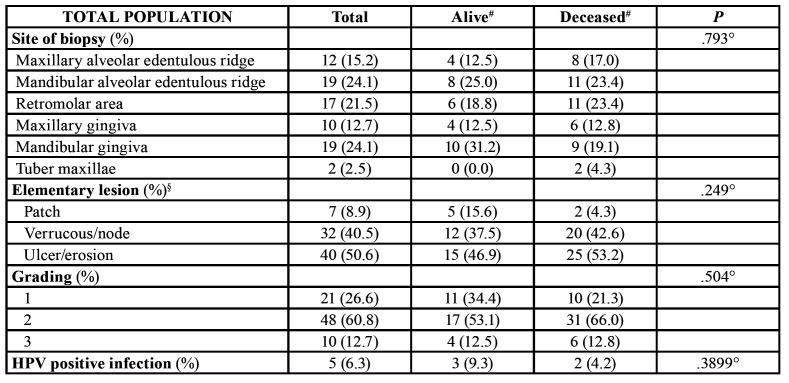
Main clinical and histopathological features of GSCCs.

**Figure 1:**
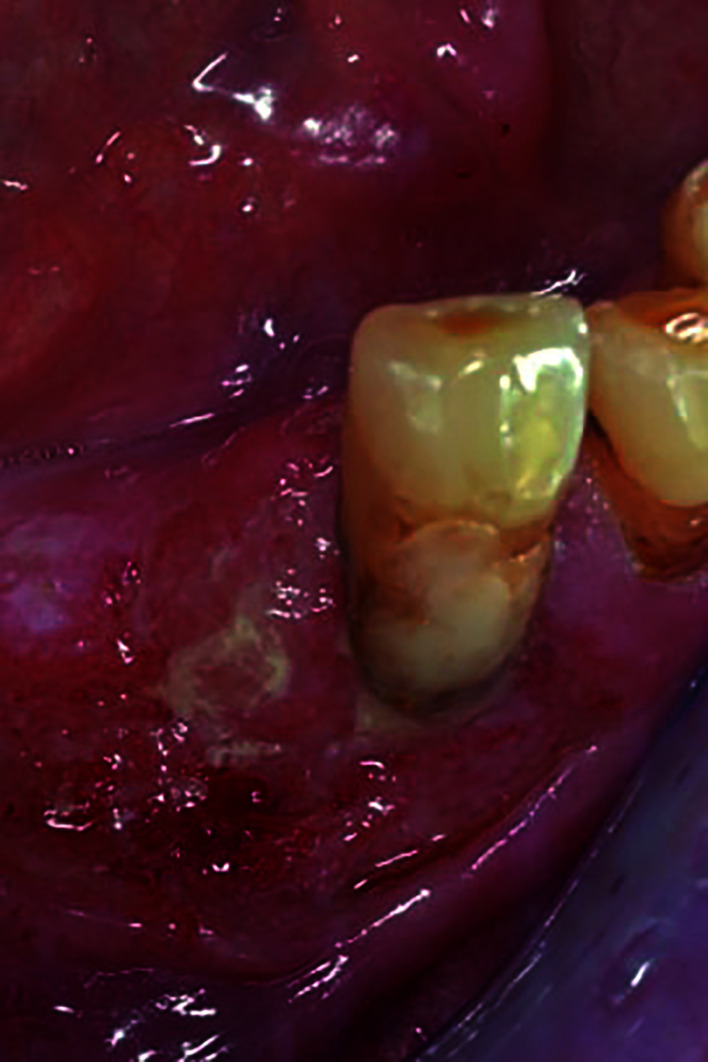
GSCC arising as non-homogeneous, ulcerative mass extending from gingiva of left mandibular premolar to the surrounding edentulous ridge.

Of the 79 GSCC, 55 (69.6%) were located in mandible, whereas 24 (31.3%) in maxilla; 34 (43.3%) were detected in the right side, 45 (56.7%) in the left side; 49 (62.02%) were detected in premolar-molar regions, and 30 (37.98%) in the incisors area. Five patients developed multiple malignancies: in detail, 2 patients experienced metachronous cancers, 1 in lower lip and 1 in side of tongue, respectively. On the other hand, 2 patients were diagnosed with synchronous cancers, 1 in fornix and 1 in buccal mucosa. Finally, 1 patient developed both a synchronous tongue cancer, and two metachronous malignancies in buccal mucosa and hard palate. Primary tumors’ size ranged from a minimum of 9 mm in one patient, to a maximum diameter of 4.2 cm in one case, with an average size of 1.7 cm. Regarding clinical appearance, 7 (8.9%) lesions were described as patches, 32 (40.5%) as either verrucous lesion or as submucosal node, with or without erosions, whereas 40 (50.6%) had ulcer or erosion as main clinical appearance (Fig. 1).

Ablative surgical resection was the main modality of treatment, and patients who presented with node positive neck disease underwent also elective neck dissection in the same way as when tumour invasion of the midline structure was observed. Adjuvant radiotherapy with local dose field of 50 to 66 Gy was used in patients with positive or close margins, vascular or perineural invasion and extracapsular spread. In detail, 23 (29.1%) patients underwent surgery alone, of whom 11 required elective neck dissection; 19 (24%) were treated with a combination of surgery, radiotherapy and chemotherapy; 12 (15.2%) underwent surgery and adjuvant radiotherapy, of whom 7 required elective neck dissection; 3 (3.8%) patients underwent surgery and chemotherapy. On the other hand, in 14 (17.7%) cases, considered not eligible for surgery, were administered either chemotherapy (5 cases), or radiotherapy (3 cases), or chemo/radiotherapy (6 cases) combined. Finally, in 8 (10.1%) cases no treatment could be provided, due to extremely poor state of health already at diagnosis. (Table 3).

The median months of follow-up was of 21.00 [8.50-61.50]. During this period, 47 (59.5%) patients died; of these 36 (45.5%) were GSCC-related deaths, while for the others it was not possible to detect the exact cause. The overall cumulative survival rate 5 years after the diagnosis was 43%, while at 10 years it was 11%.

**Figure 2:**
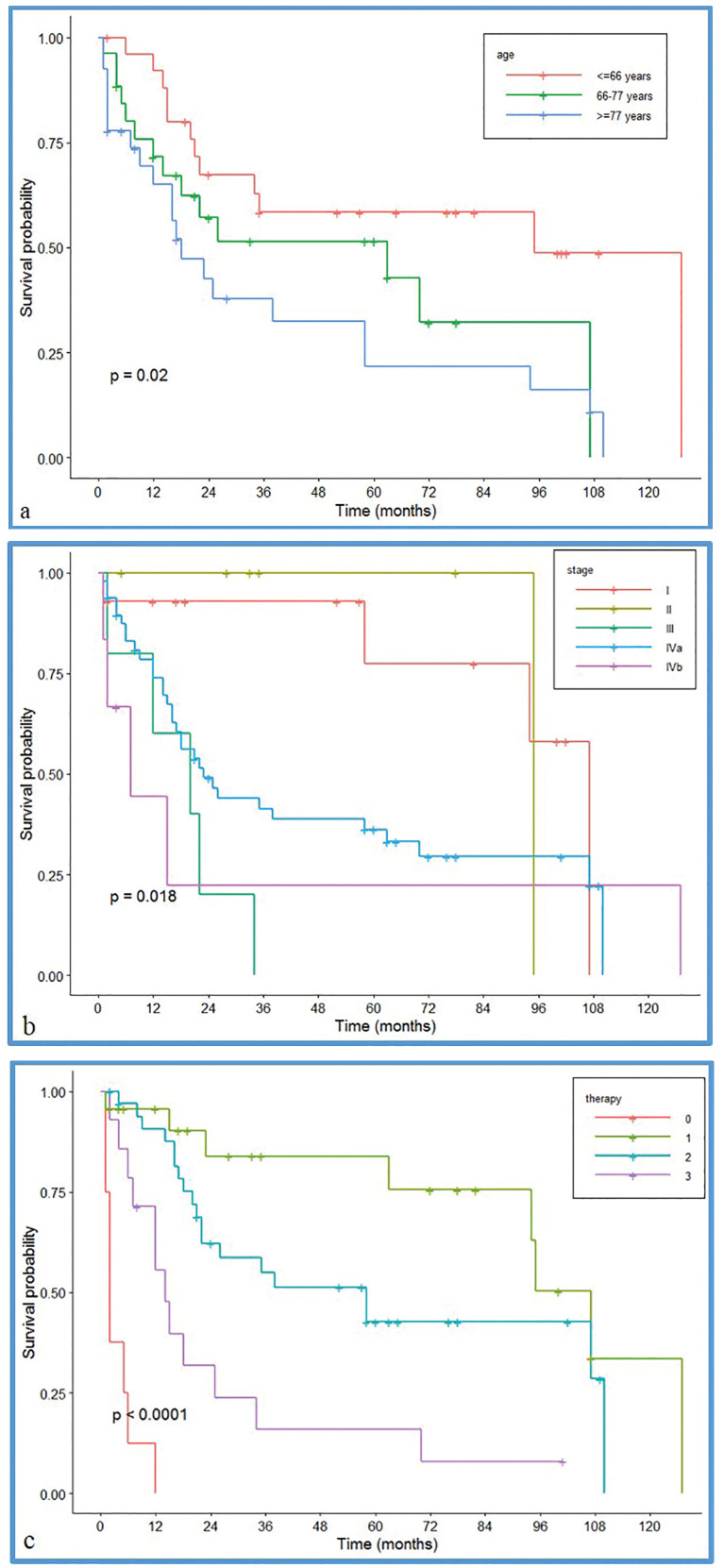
Survival curve for GSCC according to age (2a), stage (2b) and therapy (2c) (0 = none, 1= surgery, 2 = surgery plus others, 3 = others without surgery).

Survival curve for GSCC according to gender, and risk factors smoke did not show a significant difference. Moreover, univariate analysis showed that age, stage and modality of treatment could affected the survival rate (*p*=.02, *p*<.0001, and *p*=.018 respectively) (Fig. 2).

Patients with tumours at a high T and N score had a less favourable prognosis than those with lower T and N values. In particular, we described 5-year survival rates of 77% and 36% for diseases classified as stage I and stage IVa respectively (Table 4). The site and the grading of the lesions did not affect the survival rates.

The survival rates for patients treated by surgery or radiotherapy (considered separately) revealed a statistical significance between patients treated by surgery and those not treated or treated without surgery.

**Table 3:**
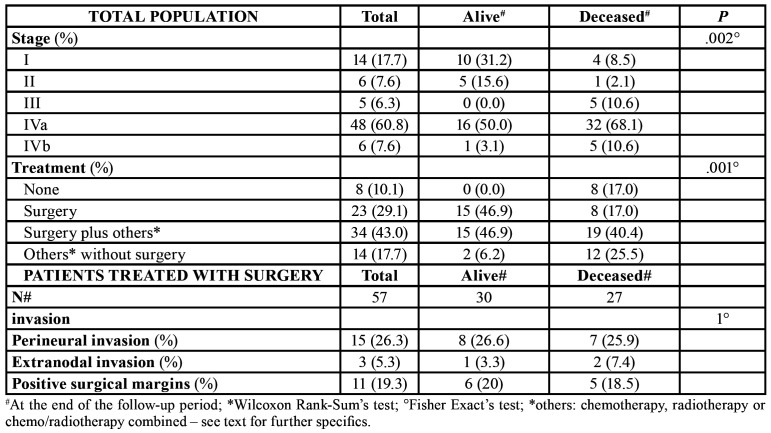
Staging and treatment of GSCCs.

**Table 4:**
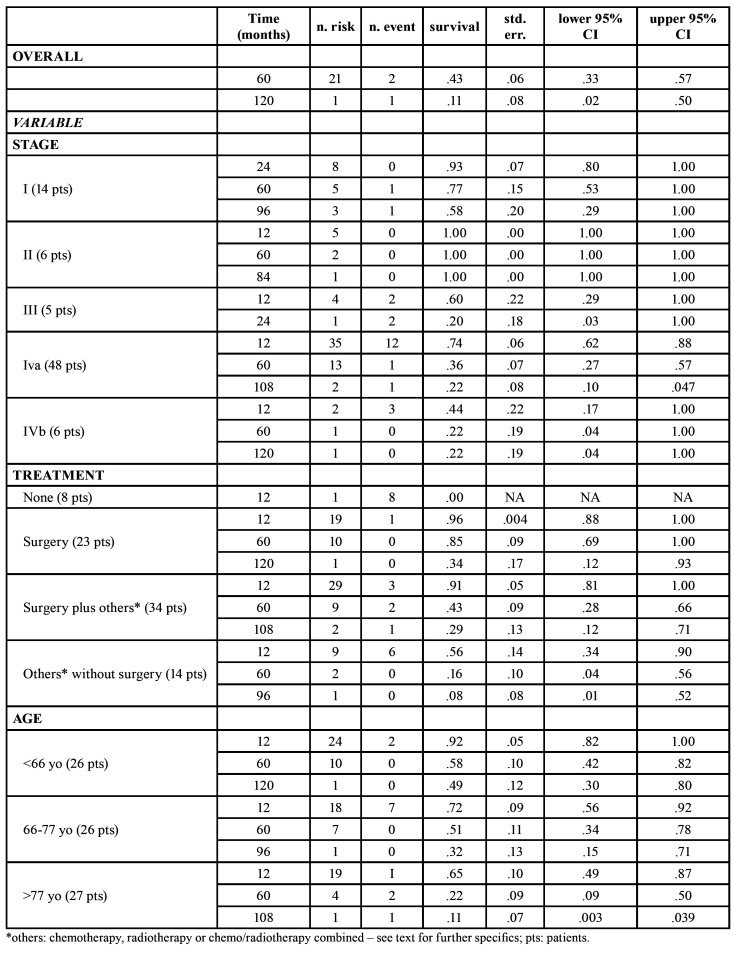
Data about survival rate, considering staging, treatment, age as variables.

## Discussion

Oral cancer is a difficult disease to control, and small improvements have been seen in the survival rate over the last decades. Moreover, to the best of our knowledge, data about GSCC are very scarce, especially from Italy.

GSCC is an insidious disease, not always having the appearance of a malignancy, ranging from an exophytic to a verrucous to an ulcerated lesion, as previously reported by these Authors (10). Such characteristic is often the cause of diagnostic delay with worsening of the prognosis, as it can simulate periodontal inflammatory pathologies or benign neoplasms (11). However, as previously reported by many Authors (12,13), as well as in a retrospective analysis of a large gingival sample by this group (14), GSCC represents by far the most common gingival malignancy to be encountered in the everyday clinical practice, for both specialists and non-specialists in oral medicine.

In this study, some factors possibly having influence on survival were analysed. Most general features of the present series were coherent with previous reports. Squamous cell carcinoma of the head and neck is commonly associated with the use of alcohol and tobacco; however, patients diagnosed with GSCC are typically not exposed to classical risk factors, as confirmed in the present series, especially for female patient (15). Moreover, in contrast with what has been usually reported about oral cancer, in the present sample the survival of smokers is on average 44 months, compared with 32 months for non-smokers.

According to the data collected in the present study, it should be necessary to pay particular attention to female patients without specific risk factors, especially when having flat lesions in the lower jaw. However, no prognostic differences emerged between males and females in the present series.

The literature reports that the GSCC most frequently involves the jaw in the female population aged over 50 years; the present study sample confirms this data with a frequency very close to 70%, but with a decidedly higher onset age (73 years for females and 67.5 for males). However, the correlation of prognosis with age seems to be quite controversial. In a case-series of 450 gingival squamous cell carcinoma, a significant influence of age distribution on prognosis was detected, with a 5-years survival rate of 61.7% among patients of less than 50 years versus 34.1% for patients above 50 years of age (15). Furthermore, a review of literature on oral carcinoma in patients twenty years of age or younger showed a striking predilection for gingiva, probably due to the high stem-cell activity of the tooth-bearing area (16). On the other hand, one of the widest population-based cohorts of OSCC available in literature, with 27717 OSCC cases scrutinized, reported lack of statistical significance between advanced staging and age, either in gingiva or in other anatomic sites (8).

Gingival SCC presents a high possibility of periosteal and bone invasion due to anatomical contiguity; this raises the risk of metastatic dissemination (17).

The course of GSCC could be determined by specific factors, above all the staging of the disease. In the present series, the set of IVa and IVb stages included almost 70% of the total patients: this could explain the strongly aggressive nature of the neoplasm probably given by the close proximity to the underlying tissue layers and therefore the easier propensity towards invasion, in turn correlated to higher and earlier mortality. A series of 334 cases of oral carcinoma, published by this group in 2008 (4), showed a disease-related mortality rate of 23.2% at five years, a Figure that is different from the current case history restricted to gingival carcinomas: the survival curve of the present sample shows that about 50% of patients died after 24 months.

To date, the treatment of GSCC remains mainly surgical, adding adjuvant radiotherapy for advanced stage disease or in patients at elevated risk of locoregional failure (4); the present data still confirmed this aspect.

Bearing in mind the limitations of a retrospective study conducted within a single-center on a relatively small sample of patients from North-West Italy, carrying a limited external validity, the present study, to the best of our knowledge, this is the biggest series of GSCC ever reported from Italy. We revealed age, stage and modality of treatment as autonomous factors in predicting survival in patients with GSCC. Carcinoma of the gingiva is an exception with regard to intraoral tumours, not apparently associated with risk factors and in close contact with the underlying tissues. It presents a high aggressiveness and the surgical therapy, frequently associated with chemotherapy and radiotherapy, is often not decisive. The similarity to periodontal lesions or benign formations leads to a diagnostic delay with a sudden worsening of the prognosis. The most worrying element is that a high mortality rate would not seem to be associated with lesions of unusual localization and therefore of more difficult investigation, but rather located in the gingival tissues which should be very recognizable in the daily clinical practice of dentists and hygienists.

With these premises, dental practitioners should play a fundamental role in intercepting simple colour changes, especially in the gingival level, and motivating the patient to undergo a more thorough investigation if a regression of the aforementioned lesions cannot be attested, especially among women in the absence of traditional risk factors.
